# L1 and epithelial cell adhesion molecules associated with gastric cancer progression and prognosis in examination of specimens from 601 patients

**DOI:** 10.1186/1756-9966-32-66

**Published:** 2013-09-16

**Authors:** Yuan-Yu Wang, Li Li, Zhong-Sheng Zhao, Yong-Xiang Wang, Zai-Yuan Ye, Hou-Quan Tao

**Affiliations:** 1 Department of Gastrointestinal Surgery, Zhejiang Provincial People’s Hospital, Hangzhou 310014, PR China; 2 Key Laboratory of Gastroenterology of Zhejiang Province, Hangzhou 310014, Zhejiang, PR China; 3 Department of Pathology, Zhejiang Provincial People’s Hospital, Hangzhou 310014, PR China

**Keywords:** Gastric carcinoma, L1CAM, EPCAM, Immunohistochemistry, Progression, Prognosis

## Abstract

**Background:**

L1 cell adhesion molecule (L1CAM) and epithelial cell adhesion molecule (EPCAM) have been implicated in the development and progression of gastric cancer. The present study investigated the clinical significance of L1CAM and EPCAM in the development, progression and prognosis of gastric cancer.

**Methods:**

Expression of L1CAM and EPCAM were examined immunochemically in 601 clinicopathologically characterized gastric cancer cases.

**Results:**

L1CAM protein was detected in 23.9% of human non-tumor mucosa samples. All samples expressed L1CAM protein at low levels. High expression of L1CAM protein was detected in 163 (27.1%) tumors. Expression of L1CAM correlated with age, tumor location, size of tumors, Lauren’s classification, depth of invasion, lymph node and distant metastases, regional lymph node stage, Tumor-Node-Metastasis (TNM) stage and prognosis. EPCAM protein was detected in 45.7% of human non-tumor mucosa samples. All samples expressed EPCAM protein at low levels. High expression of EPCAM protein was detected in 247 (41.1%) tumors. Expression of EPCAM correlated with age, tumor location, size of tumors, Lauren’s classification, depth of invasion, lymph node and distant metastases, regional lymph node stage, TNM stage and prognosis. Cumulative 5-year survival rates of patients with high expression of both L1CAM and EPCAM were significantly lower than in patients with low expression of both.

**Conclusions:**

Expression of L1CAM and EPCAM in gastric cancer was significantly associated with lymph node and distant metastasis, and poor prognosis. L1CAM and EPCAM proteins could be useful markers to predict tumor progression and prognosis.

## Introduction

Although global incidence of gastric cancer has decreased in recent years, its mortality rate in China is the highest among all tumors. The main cause of death is invasion and metastasis of tumor. Tumor invasion and metastasis is a very complicated and continuous process involving multiple steps, regulated at the molecular level by adhesion molecules, protein catabolic enzymes, cellular growth factors and various angiogenic factors. L1 cell adhesion molecule (L1CAM) is a cell adhesion molecule of the immunoglobulin superfamily of cell adhesion molecules (IgCAM), initially identified in the nervous system. Recent studies demonstrated L1CAM expression in various types of cancer, predominantly at the invasive front of tumors and metastases. Overexpression of L1CAM in normal and cancer cells increased motility, enhanced growth rate and promoted cell transformation and tumorigenicity. The epithelial cell adhesion molecule (EPCAM) is a glycoprotein of approximately 40 kD that was originally identified as a marker for carcinoma. EPCAM’s effects are not limited to cell adhesion; they include diverse processes such as signaling, cell migration, proliferation, and differentiation. Cell surface expression of EPCAM may actually prevent cell–cell adhesion.

The current study examined expression of L1CAM and EPCAM in surgical specimens of gastric carcinoma, to explore possible correlations between L1CAM and EPCAM expression and clinicopathological variables and prognosis.

## Materials and methods

### Cell culture and frozen tissues

Human gastric cancer cell lines AGS, MKN-28, BGC-823, HCG-27, SGC-7901, 9811P, MKN-45 and non-malignant gastric epithelial cell line GES-1 were obtained from Key Laboratory of Gastroenterology of Zhejiang Province (Hangzhou, China), and cultured in RPMI1640 containing 10% foetal bovine serum (FBS), 50U/ml penicillin and 50 μg/ml streptomycin. All cells were maintained at 37°C under an atmosphere of 5% CO_2_.

### Patients and frozen tissue samples

Our study included 42 patients (29 male, 13 female; mean age: 59 years; range: 30–86) collected from gastrectomy specimens from the Department of Surgery, Zhejiang Provincial People’s Hospital from January 2010 and January 2011. None of the patients were treated with radiotherapy or perioperative chemotherapy, and all had undergone total gastrectomies. Resected specimens were studied pathologically according to the criteria described in the AJCC classification (2009). There were 24 tubular adenocarcinomas, 3 papillary adenocarcinomas, 10 mucinous adenocarcinomas, 5 signet-ring cell carcinomas. Two cases were categorized as stage I, 8 as stage II, 29 as stage III, and 3 as stage IV. The study items included age, sex, tumor location, tumor size, gross (Borrmann) type, gastric wall invasion, resection margin, histological type, lymph node metastasis, vascular invasion, lymphatic invasion, and perineural invasion. Fresh samples of tumor tissue, and matched normal gastric mucosa were obtained immediately after gastric resection. The samples were dissected carefully from resected specimens by a pathologist, and immediately snap-frozen in separate vials using liquid nitrogen. These frozen specimens were stored at −80°C in a tumor bank before use.

### Patients and paraffin-embedded tissue samples

Gastric cancer tissues were collected from gastrectomy specimens of 601 patients from the Department of Surgery, Zhejiang Provincial People’s Hospital from January 1998 to January 2004. Tissues had been formalin-fixed and paraffin-embedded, and clinically and histopathologically diagnosed at the Departments of Gastrointestinal Surgery and Pathology. All patients had follow-up records over at least 5 years. The follow-up deadline was December 2008. Survival times were counted from the dates of surgery to the follow-up deadline or dates of death, which were mostly caused by carcinoma recurrence or metastasis. Ninety-two noncancerous human gastric tissues were obtained from gastrectomies of adjacent gastric cancers beyond margins >5 cm. Routine chemotherapy was given to patients with advanced-stage disease after operation, but no radiation treatment was administered to any patients included in our study.

### Real-time quantitative RT-PCR

Expressions of *L1CAM* and *EPCAM* in 42 tumor tissue samples and matched normal gastric mucosa were confirmed by RT-PCR. Total RNA was extracted by TRIzol and cDNAs were reverse-transcribed by RevertAid TM reverse transcriptase. Real-time PCR was carried out using the ABI PRISM 7700 Sequence Detection System (Applied Biosystems) at 50°C for 2 min, 95°C for 10 min, followed by 50 cycles at 95°C for 15 s, and at 60°C for 1 min. The primers for *GAPDH* (224 bp) were 5′-TGAAGGTCGGAGTCAACGG-3′ (sense) and 5′- CTGGAAGATGGTGATGGGATT-3′ (antisense). The primers for *L1CAM* (187 bp) were 5′-TGTCCTTCCCTTTACGCCAC-3′ (sense) and 5′- GACCAAGCACAGGCATACAGG-3′ (antisense). The primers for *EPCAM* (101 bp) were 5′-ATAATAATCGTCAATGCCAGTG-3′ (sense) and 5′- ATTCATTTCTGCCTTCATCAC-3′ (antisense). The expression of *GAPDH* was used to normalize that of the target genes. Each assay was done in triplicate and the average calculated. The expression level of *L1CAM/EPCAM* was expressed as 2^–ΔΔCt^, ΔCt = Ct(*Target*) – Ct(*GAPDH*).

### Tissue microarray

Blocks containing a total of 693 cases (601 cancer samples and 92 non-cancer tissue samples) were prepared as described previously [1,2].

### Immunohistochemistry

Immunohistochemical analysis was used to study altered protein expression in 92 noncancerous human gastric tissue controls and 601 human gastric cancer tissues [3,4]. In brief, slides were baked at 60°C for 2 h, followed by deparaffinization with xylene and rehydration. The sections were submerged into EDTA antigenic retrieval buffer and microwaved for antigenic retrieval, after which they were treated with 3% hydrogen peroxide in methanol to quench endogenous peroxidase activity, followed by incubation with 1% bovine serum albumin to block nonspecific binding. Sections were incubated with rabbit anti-EPCAM(Epitomics), and with mouse anti-L1CAM (Abcam), overnight at 4°C. Normal goat serum was used as a negative control. After washing, tissue sections were treated with secondary antibody. Tissue sections were then counterstained with hematoxylin, dehydrated, and mounted. Cytoplasm with L1CAM and EPCAM was stained as buffy. The degree of immunostaining was reviewed and scored independently by two observers based on the proportion of positively stained tumor cells and intensity of staining [5-7].

### Statistical analysis

All statistical analyses were performed using SPSS16.0 software. Measurement data were analyzed using Student’s *t* test, while categorical data were studied using *χ*^*2*^ or Fisher exact tests. Survival curves were estimated using the Kaplan–Meier method; the log-rank test was used to compute differences between curves. Multivariate analysis using the Cox proportional hazards regression model was performed to assess prognostic values of protein expression. Correlation coefficients between protein expression and clinicopathological findings were estimated using the Pearson correlation method. Statistical significance was set at *P* < 0.05.

## Results

### Expression of L1CAM and EPCAM mRNA in gastric tumor tissue and cell lines

**Figure 1:**
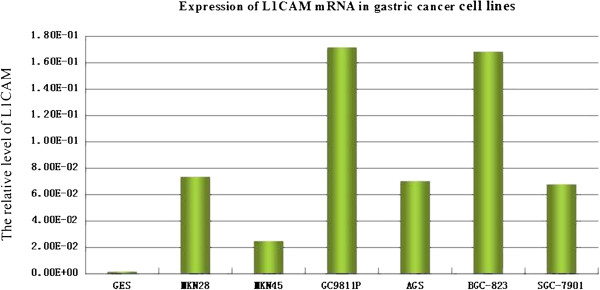
Expression of L1CAM mRNA in gastric cancer cell lines.

**Figure 2:**
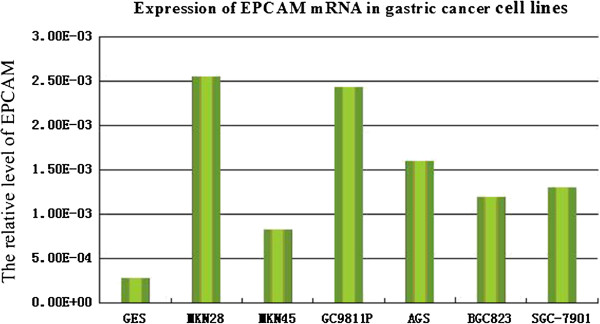
Expression of EPCAM mRNA in gastric cancer cell lines.

L1CAM and EPCAM mRNA were significantly upregulated in AGS, MKN-28, BGC-823, HCG-27, SGC-7901, 9811P and MKN-45 cell lines compared with the non-malignant gastric epithelial cell line GES-1 (P < 0.05, Figure 1, Figure 2). In 42 gastric tumor tissue samples and matched normal gastric mucosa, average expressions of *L1CAM* were 0.0403 ± 0.0069 and 0.0093 ± 0.0010, respectively, and were significant different (*t* = 2.845, *P* = 0.006). *L1CAM* was over-expressed in 25 gastric tumor tissue samples compared with matched normal gastric mucosa. In 42 gastric tumor tissue samples and matched normal gastric mucosa, the average expressions of *EPCAM* were 0.4199 ± 0.0485 and 0.1759 ± 0.0144, respectively, and were significantly different (*t* = 3.122, *P* = 0.002). *EPCAM* was over-expressed in 27 gastric tumor tissue samples compared with matched normal gastric mucosa.

### Expression of L1CAM and EPCAM in archived gastric cancer tissue and non-cancer mucosa

**Figure 3:**
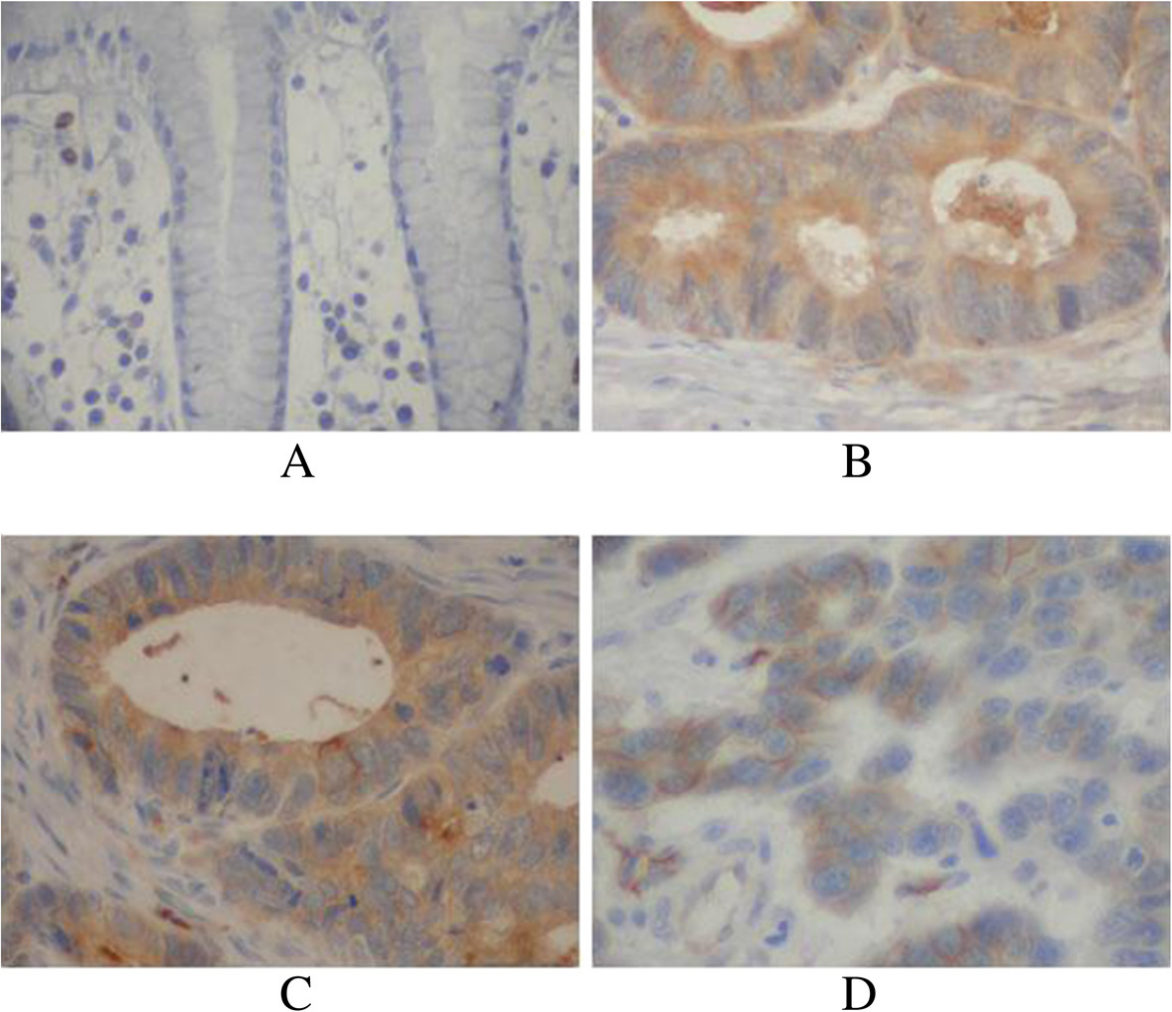
**Immunohistochemical staining for L1CAM in gastric cancer lesions (601 case) and noncancerous tissues (92 case). A**: L1CAM was negative in noncancerous tissues, **B**: L1CAM was highly expressed in well differentiated adenocarcinoma, **C**: L1CAM was highly expressed in moderately differentiated adenocarcinoma, **D**: L1CAM was highly expressed in poorly differentiated adenocarcinoma.

L1CAM protein was detected in 22/92 (23.9%) human non-tumor mucosa samples; all samples expressed L1CAM protein at low levels. High L1CAM protein expression was detected in 163 (27.1%) tumors. L1CAM was localized mainly in the cytoplasm of primary cancer cells (Figure 3).

**Figure 4:**
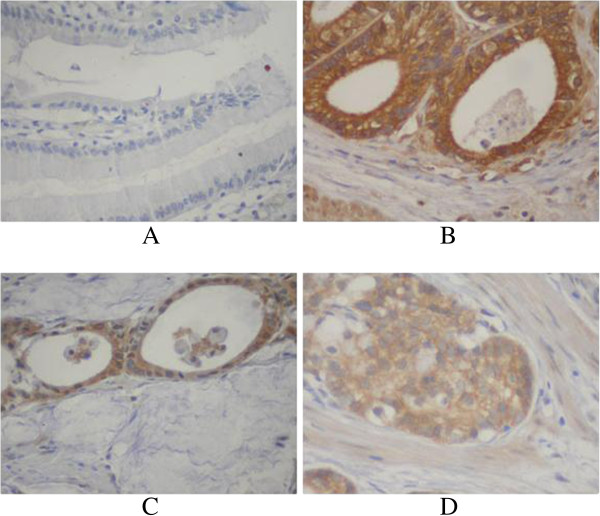
**Immunohistochemical staining for EPCAM in gastric cancer lesions (601 case) and noncancerous tissues (92 case). A**: EPCAM was negative in noncancerous tissues, **B**: EPCAM was highly expressed in well differentiated adenocarcinoma, **C**: EPCAM was highly expressed in moderately differentiated adenocarcinoma, **D**: EPCAM was highly expressed in poorly differentiated adenocarcinoma.

EPCAM protein was detected in 42/92 (45.7%) human non-tumor mucosa samples; all samples expressed EPCAM protein at a low level. High EPCAM protein expression was detected in 247 (41.1%) tumors, EPCAM was localized mainly in the cytoplasm of primary cancer cells (Figure 4).

### L1CAM and EPCAM overexpression and clinicopathological features

Expression of L1CAM correlated with age, tumor location, tumor size, Lauren’s classification, depth of invasion, lymph node and distant metastases, regional lymph node stage and TNM stage (*P* < 0.05). L1CAM expression did not correlate with sex, differentiation, or histological classification (*P*>0.05; Table 1).

**Table 1:** Relationship of L1CAM expression with pathological parameters of tumor

**Clinical parameters**	**L1CAM**			
	**Low**	**High**	**t/χ**^**2**^**/r**	**P**
Age(yrs)	57.86 ± 11.88	61.20 ± 11.85	3.065	0.002
Gender			3.386	0.066
Male	321 (75.0%)	107 (25.0%)		
Female	117 (67.6%)	56 (32.4%)		
Location			13.39	0.001
Proximal	54 (64.3%)	30 (35.7%)		
Middle	150 (67.3%)	73 (32.7%)		
Distal	234 (79.6%)	60 (20.4%)		
Size			26.99	0.0001
<5 cm	283 (80.9%)	67 (19.1%)		
≥5 cm	155 (61.8%)	96 (38.2%)		
Lauren classification			94.92	0.0001
Intestinal	271 (90.6%)	28 (9.4%)		
Diffuse	167 (55.3%)	135 (44.7%)		
Histology			5.623	0.131
Papillary adenocarcinoma	26 (89.7%)	3 (10.3%)		
Tubular adenocarcinoma	317 (72.2%)	122 (27.8%)		
Mucinous adenocarcinoma	29 (78.4%)	8 (21.6%)		
Signet-ring cell carcinoma	66 (68.8%)	30 (31.2%)		
Histologic differentiation			7.67	0.053
Well	17 (100%)	0 (0.0%)		
Moderately	129 (73.7%)	46 (26.3%)		
Poorly	290 (71.3%)	117 (28.7%)		
Others	2 (100.0%)	0 (0.0%)		
Invasion depth			46.55	0.0001
T1	72 (90.0%)	8 (10.0%)		
T2	123 (87.2%)	18 (12.8%)		
T3	222 (65.7%)	116 (34.3%)		
T4	21 (50.0%)	21 (50.0%)		
TNM stages			85.48	0.0001
I	119 (93.7%)	8 (6.3%)		
II	121 (89.6%)	14 (10.4%)		
III	141 (61.0%)	90 (39.0%)		
IV	57 (52.8%)	51 (47.2%)		
Lymphatic metastasis			43.59	0.0001
No	195 (88.6%)	25 (11.4%)		
Yes	243 (63.8%)	138 (36.2%)		
Regional lymph nodes			59.62	0.0001
PN0	195 (88.6%)	25 (11.4%)		
PN1	142 (71.7%)	56 (28.3%)		
PN2	79 (58.5%)	56 (41.5%)		
PN3	22 (45.8%)	26 (54.2%)		
Distant metastasis			15.376	0.0001
No	387 (75.9%)	123 (24.1%)		
Yes	51 (56.0%)	40 (44.0%)		

Expression of EPCAM correlated with age, tumor location, tumor size, Lauren’s classification, depth of invasion, lymph node and distant metastases, regional lymph node stage and TNM stage (*P* < 0.05). EPCAM expression did not correlate with sex, differentiation, or histological classification (*P* > 0.05; Table 2).

**Table 2:** Relationship of EPCAM expression with pathological parameters of tumor

**Clinical parameters**	**EPCAM**			
	**Low**	**High**	**t/χ**^**2**^**/r**	**P**
Age(yrs)	56.85 ± 11.4	61.51 ± 12.22	4.787	0.0001
Gender			0.805	0.370
Male	257 (60.0%)	171 (40.0%)		
Female	97 (56.1%)	76 (43.9%)		
Location			10.37	0.006
Proximal	37 (44.0%)	47 (56.0%)		
Middle	130 (58.3%)	93 (41.7%)		
Distal	187 (63.6%)	107 (36.4%)		
Size			40.47	0.0001
<5 cm	244 (69.7%)	106 (30.3%)		
≥5 cm	110 (43.8%)	141 (56.2%)		
Lauren classification			198.1	0.0001
Intestinal	261 (87.3%)	38 (12.7%)		
Diffuse	93 (30.8%)	209 (69.2%)		
Histology			3.136	0.371
Papillary adenocarcinoma	20 (69.0%)	9 (31.0%)		
Tubular adenocarcinoma	254 (57.9%)	185 (42.1%)		
Mucinous adenocarcinoma	19 (51.4%)	18 (48.6%)		
Signet-ring cell carcinoma	61 (63.5%)	35 (36.5%)		
Histologic differentiation			6.323	0.097
Well	12 (70.6%)	5 (29.4%)		
Moderately	113 (64.6%)	62 (35.4%)		
Poorly	227 (55.8%)	180 (44.2%)		
Others	2 (100.0%)	0 (0.0%)		
Invasion depth			107.1	0.0001
T1	73 (91.2%)	7 (8.8%)		
T2	113 (80.1%)	28 (19.9%)		
T3	160 (47.3%)	178 (52.7%)		
T4	8 (19.0%)	34 (81.0%)		
TNM stages			201.6	0.0001
I	119 (93.7%)	8 (6.3%)		
II	116 (85.9%)	19 (14.1%)		
III	99 (42.9%)	132 (57.1%)		
IV	20 (18.5%)	88 (81.5%)		
Lymphatic metastasis			119.1	0.0001
No	193 (87.7%)	27 (12.3%)		
Yes	161 (42.3%)	220 (57.5%)		
Regional lymph nodes			182.6	0.0001
PN0	193 (87.7%)	27 (12.3%)		
PN1	118 (59.6%)	80 (40.4%)		
PN2	42 (31.1%)	93 (68.9%)		
PN3	1 (2.1%)	47 (97.9%)		
Distant metastasis			53.42	0.0001
No	332 (65.1%)	178 (34.9%)		
Yes	22 (24.2%)	69 (75.8%)		

### Correlation between L1CAM and EPCAM expression and patient prognosis

As TNM stage, lymph node and distant metastasis are used as prognostic factors for gastric cancer [8], we further analyzed the correlation between L1CAM/EPCAM expression and patient prognosis according to Lauren classification, TNM stage and regional lymph nodes.

**Figure 5:**
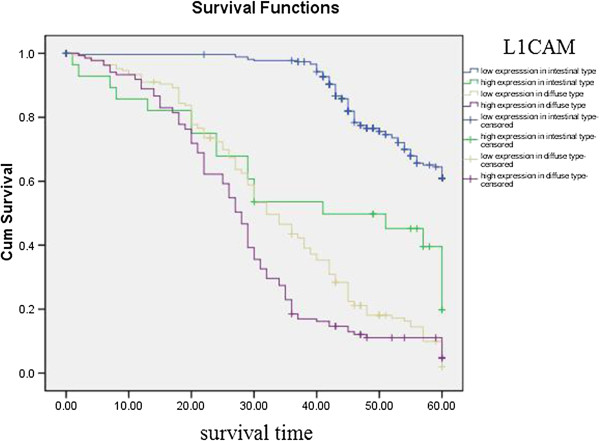
Kaplan-Meier curves with univariate analyses (log-rank) for patients with low L1CAM expression versus high L1CAM expression tumors according to Lauren classification.

**Figure 6:**
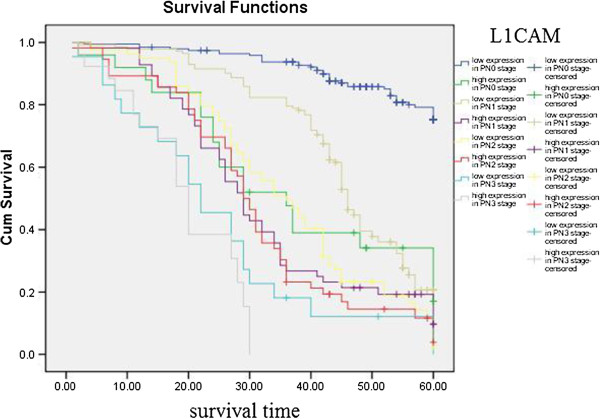
Kaplan-Meier curves with univariate analyses (log-rank) for patients with low L1CAM expression versus high L1CAM expression tumors according to regional lymph nodes.

**Figure 7:**
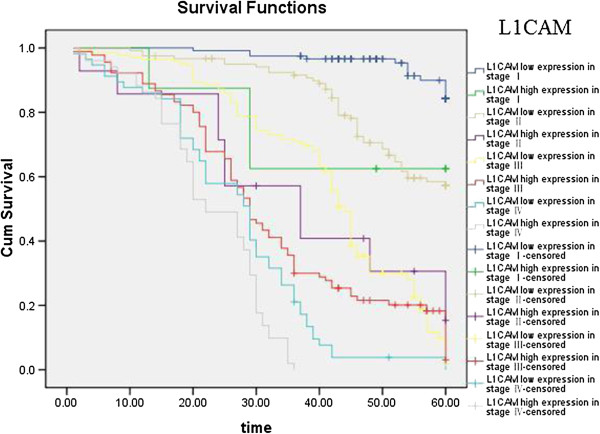
Kaplan-Meier curves with univariate analyses (log-rank) for patients with low L1CAM expression versus high L1CAM expression tumors according to TNM stage.

Kaplan–Meier curves with univariate analyses (log-rank) for patients with low L1CAM expression versus high L1CAM expression tumors according to Lauren classification, showed significant differences (Table 3, Figure 5), as did Kaplan–Meier curves with univariate analyses (log-rank) for patients with low L1CAM expression versus high L1CAM expression tumors according to regional lymph nodes. Cumulative 5-year survival rates for patients with low L1CAM were significantly higher than in patients with high L1CAM expression among those in PN0 and PN1 stages (Table 3, Figure 6). Kaplan–Meier curves with univariate analyses (log-rank) for patients with low L1CAM expression versus high L1CAM expression tumors according to TNM stage, showed cumulative 5-year survival rates for patients with low L1CAM were significantly higher than in patients with high L1CAM expression among those in stage I , stage II and stage III (Table 3, Figure 7).

**Table 3:** Correlation between the expression of L1CAM and prognosis

	**Low expression of L1CAM**	**High expression of L1CAM**	**χ**^**2**^	***P***
Intestinal-type	68.3%	35.7%	22.83	0.001
Diffuse-type	10.8%	8.9%	7.86	0.005
PN0	79.5%	28.0%	59.06	0.0001
PN1	29.6%	16.1%	19.1	0.0001
PN2	12.7%	10.7%	2.47	0.116
PN3	9.1%	0%	2.16	0.14
Stage I	89.1%	62.5%	6.95	0.008
Stage II	62.0%	33.3%	21.86	0.0001
Stage III	18.6%	15.9%	8.45	0.004
Stage IV	3.5%	0%	7.003	0.08

**Figure 8:**
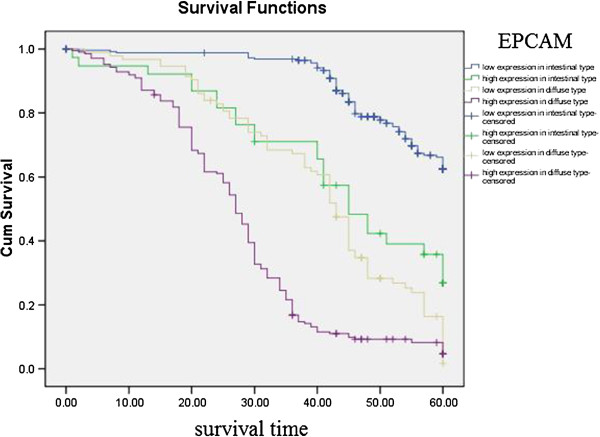
Kaplan-Meier curves with univariate analyses (log-rank) for patients with low EPCAM expression versus high EPCAM expression tumors according to Lauren classification.

**Figure 9:**
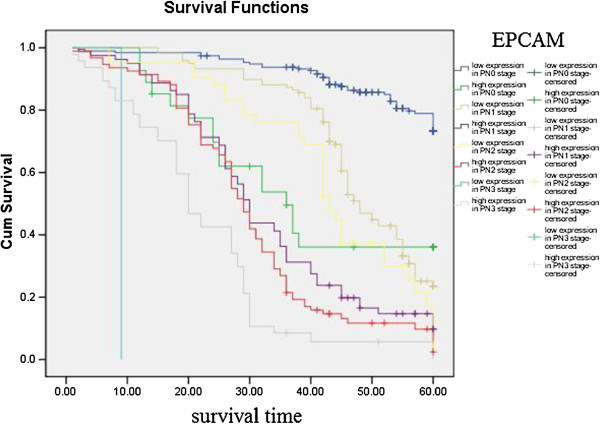
Kaplan-Meier curves with univariate analyses (log-rank) for patients with low EPCAM expression versus high EPCAM expression tumors according to regional lymph nodes.

**Figure 10:**
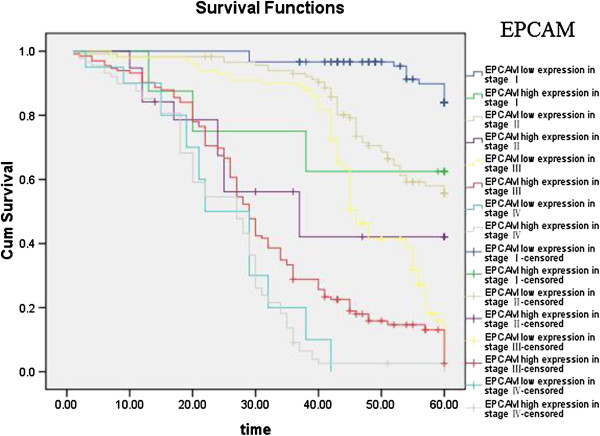
Kaplan-Meier curves with univariate analyses (log-rank) for patients with low EPCAM expression versus high EPCAM expression tumors according to TNM stage.

Kaplan–Meier curves with univariate analyses (log-rank) for patients with low EPCAM expression versus high EPCAM expression tumors according to Lauren classification and regional lymph nodes showed cumulative 5-year survival rates for patients with low EPCAM was significantly higher than for patients with high EPCAM expression (Figures 8, 9; Table 4). Kaplan–Meier curves with univariate analyses (log-rank) for patients with low EPCAM expression versus high EPCAM expression tumors according to TNM stage, showed cumulative 5-year survival rates for patients with low EPCAM were significantly higher than in patients with high EPCAM expression among those in stage I , stage II and stage III (Table 4, Figure 10).

**Table 4:** Correlation between the expression of EPCAM and prognosis

	**Low expression of EPCAM**	**High expression of EPCAM**	**χ**^**2**^	***P***
Intestinal-type	6.9.7%	34.2%	29.15	0.001
Diffuse-type	12.9%	8.6%	37.11	0.001
PN0	78.2%	40.7%	35.77	0.001
PN1	33.1%	15.0%	37.72	0.001
PN2	19.0%	8.6%	17.31	0.001
PN3	4.3%	0%	3.21	0.073
Stage I	89.1%	62.5%	4.89	0.027
Stage II	60.3%	47.4%	7.648	0.006
Stage III	22.2%	12.9%	35.58	0.0001
Stage IV	0%	2.3%	0.268	0.605

Factors with possible prognostic effects in gastric carcinoma were analyzed by Cox regression analysis. The study revealed that depth of invasion (*P*=0.007), lymph node (*P* = 0.009) and distant metastasis (*P* = 0.01), TNM stage (*P* = 0.008), expression of L1CAM (*P* = 0.007), and of EPCAM (*P* = 0.009) were independent prognostic factors in patients with gastric carcinoma. However, the location of the tumor, tumor size, histological type, differentiation, and vessel invasion had no prognostic value.

### Association among expression of L1CAM and EPCAM

Three hundred and sixteen gastric cancer cases had low expression of both L1CAM and EPCAM; 125 gastric cancer cases had high expression of both L1CAM and EPCAM. L1CAM and EPCAM expressions were significantly correlated (χ^2^ = 117.0, *P* = 0.0001). Cumulative 5-year survival rates of patients with high expression of both L1CAM and EPCAM were significantly lower than in patients with low expression of both (60.1% vs 11.2%, χ^2^ = 261.52, *P* = 0.0001).

## Discussion

Tumor invasion and metastasis is a very complicated and continuous process involving multiple steps, regulated at the molecular level by adhesion molecules, protein catabolic enzymes, cellular growth factors and various angiogenic factors.

The L1 cell adhesion molecule (L1CAM) belongs to the immunoglobulin superfamily and was originally identified in the nervous system. Recent studies demonstrated L1CAM expression in various types of cancer, predominantly at the invasive front of tumors and in metastases, which indicates its involvement in advanced stages of tumor progression. Overexpression of L1CAM in normal and cancer cells increases motility, enhances growth rate and promotes cell transformation and tumorigenicity. Moreover, L1CAM expression in tumor cells conferred the capacity to form metastases [9,10]. L1CAM was overexpressed in esophageal adenocarcinoma [11], pancreatic cancer [12,13], colorectal cancer [14], gallbladder carcinoma [15], extrahepatic cholangiocarcinoma [16], gastric cancer [17], and cholangiocarcinoma [18], notably at the invasive front of the tumors. Our study indicated L1CAM protein was highly expressed in 163 (27.1%) tumors. L1CAM was localized mainly in the cytoplasm of primary cancer cells. The present study shows L1CAM expression in tumors correlated with histologic grade, Lauren’s classification, depth of invasion, lymph node and distant metastases, and prognosis. Kodera detected L1CAM expression in 15 of 72 pT3-stage gastric cancer specimens with L1CAM expression more common in intestinal cancer types. Prognosis of patients with L1CAM+ cancer was significantly inferior, particularly among those with diffuse-type cancers [17]. Positive L1CAM expression was significantly correlated with histological grade, lymph node involvement, distant metastasis and survival [19]. Positive L1CAM expression in pancreatic ductal adenocarcinoma was associated with node involvement, vascular invasion, perineural invasion, higher degree of pain, and poor survival [13]. L1CAM expression in gallbladder carcinomas was significantly associated with high histologic grade, advanced pathologic T stage and clinical stage, and positive venous/lymphatic invasion. Multivariate analyses showed that L1CAM expression and clinical stage were independent risk factor for disease-free survival [15]. High expression of L1CAM in extrahepatic cholangiocarcinoma was detected at the invasive front of tumors and was significantly associated with perineural invasion. Univariate analysis indicated that various prognostic factors such as histologic grade 3, advanced pathologic T stage and clinical stage, perineural invasion, nodal metastasis, and high L1CAM expression were risk factors predicting poorer patient survival. Multivariate analyses using Cox’s proportional hazards model showed that high L1CAM expression and nodal metastasis were independent risk factors for patient death [16]. Aberrant L1CAM expression in colorectal cancer correlated with advanced stage and presence of lymph node and distant metastases [20].

Epithelial cell adhesion molecule (EPCAM) is overexpressed in most solid cancers and it has recently been identified as a cancer stem cell marker [21]. EPCAM overexpression was observed in esophageal cancer [22], pancreatic cancer and ampullary cancer samples [23], colon cancers, gastric cancers, prostate cancers, and lung cancers [24]. Our study showed high expression of EPCAM protein was detected in 247(41.1%) gastric cancers. Further study revealed EPCAM expression correlated with age, tumor location, tumor size, Lauren’s classification, depth of invasion, lymph node and distant metastases, regional lymph node stage, TNM stage and prognosis. EPCAM was found to be overexpressed in gastric cancer tissues [25]. Patients with EPCAM expression had a significantly better 10-year survival than patients with no EPCAM expression: 42% vs 22%. Loss of EPCAM expression identifies aggressive tumors, especially in patients with stage I and II disease [26]. The high EPCAM expression group showed significantly good prognosis in both overall survival and disease-free survival compared with the low-expression group. In multivariate analysis, EPCAM expression was an independent prognostic factor, along with histology and lymph node metastasis [27]. EPCAM overexpression correlated with shorter overall survival among patients with ampullary cancer and advanced stage pancreatic cancer, and was found to correlate with tumor stage of ampullary cancer [23]. EPCAM expression in human esophageal cancer correlated with tumor depth, stage, blood-vessel invasion and infiltrative growth pattern. Survival rates for patients with tumors with high EPCAM expression was significantly higher than for patients with tumors with low EPCAM expression [22].

The most important prognostic factor for gastric cancer is lymph node metastasis [28,29]. We did not find literature about the relationship between expression of EPCAM/L1CAM and prognosis of patients according to regional lymph nodes. We therefore analyzed the relationship between expression of EPCAM/L1CAM and prognosis of patients with gastric cancer according to regional lymph nodes. Cumulative 5-year survival rates for patients with low L1CAM was significantly higher than for patients with high L1CAM expression in PN1. Cumulative 5-year survival rates for patients with low EPCAM was significantly higher than for patients with high EPCAM expression in PN0, in PN1, and in PN2. Lauren classification is helpful from an epidemiological standpoint [30], Lauren classification has been useful in evaluating the natural history of gastric carcinoma, especially with regard to incidence trends, clinicopathological correlations, and etiological precursors [31]. We investigated the intestinal and diffuse types in our study. Patients with the mixed and unclassified types were not investigated because we did not have these patients. We analyze the relationship between the expression of EPCAM/L1CAM and the prognosis of patients with gastric cancer according to Lauren classification. The cumulative 5-year survival rates for both the low-L1CAM expression group and the low-EPCAM expression group were higher than for their respective high-expression groups in intestinal-type gastric cancer and diffuse-type gastric cancer. There was no literature about the relationship between expression of EPCAM/L1CAM and prognosis of patients according to Lauren classification. To avoid biasing the prognostic value of EPCAM/L1CAM by tumor stage, we analyzed the relationship between expression of EPCAM/L1CAM and prognosis of patients with gastric cancer according to TNM stage. The cumulative 5-year survival rates for both the low-L1CAM expression group and the low-EPCAM expression group were higher than for their respective high-expression groups in stages I–III.

Our study suggests that overexpression of EPCAM and L1CAM is common in gastric cancer, and plays an important role in the progression and metastasis of gastric cancer. These results imply that EPCAM and L1CAM could be useful prognostic and survival indicators. Our study provides a basis for the development of a novel biomarker for the diagnosis and prognosis of gastric cancer.

## Competing interests

The authors declared that they have no competing interests.

## Authors’ contributions

Z-SZ, Z-YY and Y-YW design the study, LL, Y-XW, and H-QT carried out the Realtime quantitative RT-PCR and immunohistochemistry, Y-SS drafted the manuscript. All authors read and approved the final manuscript.
